# Limited Phenotypic Effects of Selectively Augmenting the SMN Protein in the Neurons of a Mouse Model of Severe Spinal Muscular Atrophy

**DOI:** 10.1371/journal.pone.0046353

**Published:** 2012-09-27

**Authors:** Andrew J-H. Lee, Tomoyuki Awano, Gyu-Hwan Park, Umrao R. Monani

**Affiliations:** 1 Department of Pathology and Cell Biology, Columbia University, New York, New York, United States of America; 2 Department of Neurology, Columbia University, New York, New York, United States of America; 3 Department of Neuropharmacology, Kyungpook National University, Daegu, South Korea; 4 Center for Motor Neuron Biology and Disease, Columbia University, New York, New York, United States of America; University of Edinburgh, United Kingdom

## Abstract

The selective vulnerability of motor neurons to paucity of Survival Motor Neuron (SMN) protein is a defining feature of human spinal muscular atrophy (SMA) and indicative of a unique requirement for adequate levels of the protein in these cells. However, the relative contribution of SMN-depleted motor neurons to the disease process is uncertain and it is possible that their characteristic loss and the overall SMA phenotype is a consequence of low protein in multiple cell types including neighboring spinal neurons and non-neuronal tissue. To explore the tissue-specific requirements for SMN and, especially, the salutary effects of restoring normal levels of the protein to neuronal tissue of affected individuals, we have selectively expressed the protein in neurons of mice that model severe SMA. Expressing SMN pan-neuronally in mutant mice mitigated specific aspects of the disease phenotype. Motor performance of the mice improved and the loss of spinal motor neurons that characterizes the disease was arrested. Proprioceptive synapses on the motor neurons were restored and defects of the neuromuscular junctions mitigated. The improvements at the cellular level were reflected in a four-fold increase in survival. Nevertheless, mutants expressing neuronal SMN did not live beyond three weeks of birth, a relatively poor outcome compared to the effects of ubiquitously restoring SMN. This suggests that although neurons and, in particular, spinal motor neurons constitute critical cellular sites of action of the SMN protein, a truly effective treatment of severe SMA will require restoring the protein to multiple cell types including non-neuronal tissue.

## Introduction

Spinal muscular atrophy (SMA) is a common, autosomal recessive neuromuscular disease caused by mutations in the Survival of Motor Neurons 1 *(SMN1)* gene and thus loss of protein from the gene [1]–[3]. An almost identical homologue, *SMN2*, which is always found in SMA patients, is unable to fully compensate for loss of *SMN1* because of a translationally silent nucleotide change that alters the splicing pattern of the *SMN2* gene, rendering most of its transcripts devoid of exon 7 [4], [5]. Relatively few transcripts remain full-length SMN. The SMNΔ7 protein which derives from the transcript lacking exon 7 is unstable and rapidly degraded. Still, the invariable presence of *SMN2* in all patients ensures ubiquitous low levels of the SMN protein. SMN protein levels and, consequently, SMA severity generally correlate with *SMN2* copy number [6], [7]. Nevertheless, a common characteristic of SMA patients, from the severely affected to the mildly disposed, is evidence of early death or dysfunction of the spinal motor neurons [8], [9]. This is true of animal models as well [10]–[13], suggesting that motor neurons are particularly sensitive to SMN paucity and the SMA phenotype mostly a consequence of motor neuron loss. These assumptions have been explored in numerous studies of animal models, but the interpretations of the results continue to be debated. Transgenic over-expression of SMN in the nervous systems of SMA mice effected almost complete rescue of the disease phenotype [14], but the degree of rescue attributable to expression within the motor neurons could not be unequivocally determined owing to the expression of the SMN transgene in all neurons as well as skeletal muscle, a second likely contributor to the SMA phenotype [15]–[17]. In fly models of SMA, neuronal expression of SMN brought about a decidedly modest rescue of the disease phenotype and, accordingly, knockdown of the protein in neurons of healthy flies had a more muted effect than expected of a disease resulting primarily from motor neuron dysfunction [10], [18]. More selective restoration of SMN to a sub-population of spinal motor neurons of model mice rescued the neuromuscular phenotype but had little effect on the neonatal lethality observed in severe SMA models [19], suggestive of the contributing effects of other cell types to the overall disease phenotype. A more recent, independent study that reported similar results lends credence to this notion [20]. One possibility, particularly in light of reduced proprioceptive synapses on SMA motor neurons, [21]–[23] is a primary or, perhaps, an exacerbating effect of diseased Ia sensory neurons.

To further define the key cellular sites of action of the SMN protein in causing the SMA phenotype and, in particular, to investigate the role of the spinal neurons in the disease process and as therapeutic targets, we have restored SMN selectively to all CNS neurons of severe SMA model mice. In this study, we demonstrate that doing so rescued early motor behavior defects, arrested the characteristic loss of spinal motor neurons, restored proprioceptive synapses on the motor neurons and mitigated pre- and post-synaptic abnormalities of the neuromuscular junctions (NMJs). Nevertheless, phenotypic rescue was far from complete, indicative of non-neuronal cell types that are a) critical determinants of the overall SMA phenotype and, b) will likely have to be targeted to achieve maximum therapeutic benefit. We conclude that the most effective SMN-based treatments for SMA will require restoring the protein to multiple cell types within the organism.

## Results

### Selective induction of an Smn “rescue” allele in neuronal tissue of transgenic mice

**Figure 1:**
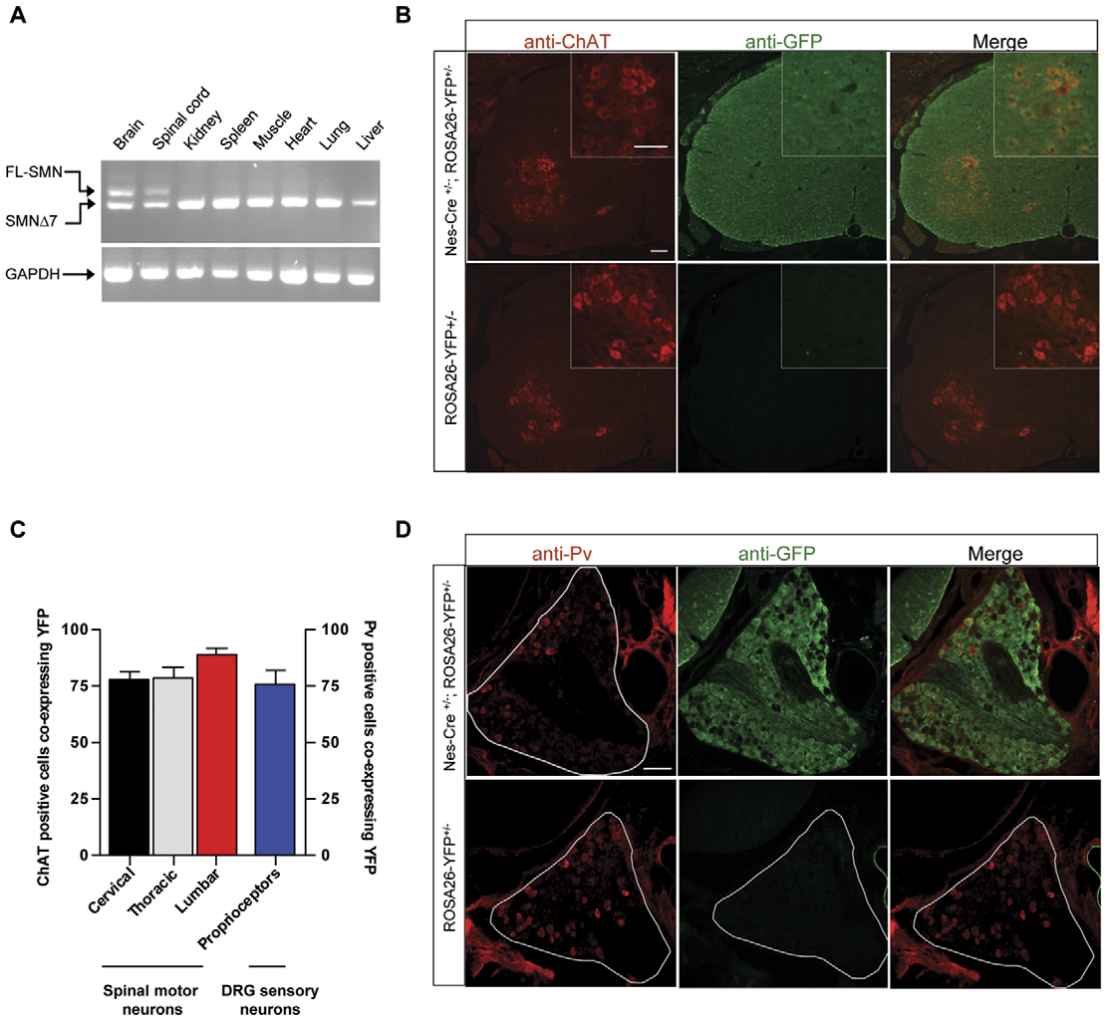
Selective activation of the *Smn* rescue allele in nervous tissue of transgenic mice. (**A**) RT-PCR analysis of tissue from an adult (PND32) double transgenic *(Nes-Cre;Smn^Res/+^)* mouse. The presence of the FL-SMN transcript specifically in brain and spinal cord is evidence of selective activation of the inducible *Smn* allele in nervous tissue. The persistence of the SMNΔ7 transcript is expected in these two tissues if even a low proportion of cells retain the un-recombined *Smn* allele. (**B**) Lumbar spinal cord sections from ROSA-YFP reporter mice with or without the Nes-Cre transgene, dually labeled with antibodies against the GFP and ChAT proteins. Widespread YFP expression including within ChAT positive motor neurons (inset) of only the double transgenic mice is consistent with pan-neuronal Cre expression from the Nes-Cre transgene. (**C**) Quantification of ChAT positive spinal motor neurons and parvalbumin (Pv) positive DRG proprioceptive sensory neurons co-expressing the YFP protein as a measure of the efficiency of Nes-Cre-driven recombination of floxed alleles. (**D**) DRGs from the lumbar spinal cord of ROSA-YFP reporter mice with or without the Nes-Cre transgene dual labeled with antibodies against Pv and GFP. YFP expression in the DRGs of only the double transgenic mice is evidence of the activation of floxed alleles within DRG proprioceptive sensory neurons. Note: Scale bars in panel (B) – 100 µm, panel (D) – 100 µm.

To selectively restore the SMN protein to neurons of SMA model mice, we tested the ability of a Cre recombinase transgene, under the control of a rat Nestin enhancer, to activate the expression of an inducible *Smn* rescue allele. Mice harboring the Nestin-Cre (Nes-Cre) transgene have been previously described [24]–[26], and the transgene shown to bring about robust recombination of floxed alleles in neurons as early as embryonic day 9.5 [27]. The *Smn* rescue allele was originally generated to determine the temporal requirements for the SMN protein and includes a hybrid genomic cassette consisting of murine *Smn* exon 7 in a reverse (3′→5′) orientation flanked by exons 7 and 8 of the *SMN2* gene [28]. The cassette was placed downstream of the endogenous murine *Smn* exon 6 and results in predominantly exon 7 deleted transcripts from the mouse/human hybrid allele and thus negligible functional SMN protein [28]. Following Cre mediated recombination, murine *Smn* exon 7 lying within the cassette is restored to its normal (5′→3′) orientation, ensuring wild-type levels of FL-SMN transcript and thus normal protein levels from the rescue allele. To test the ability of the Nestin-Cre transgene to selectively activate the rescue allele in nervous tissue, we generated double transgenic *Nes-Cre;Smn^Res/+^* mice. Multiple tissues from the mice were dissected and RNA extracted for analysis of SMN transcripts. Following RT-PCR analysis we found that FL-SMN transcript expression was restricted to brain and spinal cord tissue, whereas the transcript lacking exon 7 (SMNΔ7) continued to be expressed in all tissues indicating selective recombination and thus activation of the *Smn* rescue allele in nervous tissue (Fig. 1A).

To obtain a quantitative estimate of the level of recombination brought about specifically in the motor neurons of *Nes-Cre;Smn^Res/+^* animals, we substituted the *Smn* rescue allele with a ROSA26-flox-STOP-flox-YFP reporter transgene [29]. Motor neurons of ROSA26-flox-STOP-flox-YFP (ROSA-YFP) mice with or without the Nestin-Cre transgene were examined for evidence of YFP expression. We found more than 78% of large choline acetyltransferase (ChAT) positive motor neurons along the rostro-caudal length of the spinal cord to be positive for YFP, indicative of relatively robust Nestin-Cre mediated recombination of floxed alleles (Fig. 1B, C). Furthermore and consistent with prior reports of the pan-neuronal pattern of expression of the Nes-Cre transgene [24]–[27], the entire spinal cords, including dorsal root ganglion (DRG) sensory neurons, and brains of *Nes-Cre;ROSA-YFP* mice were found to express YFP protein (Fig. 1B, D and data not shown). To estimate numbers of proprioceptive neurons in which the Nes-Cre transgene was active, DRGs were co-stained for parvalbumin (Pv), a marker of all proprioceptive sensory neurons [30], [31], and YFP. Greater than 75% of Pv positive proprioceptors in *Nes-Cre;ROSA-YFP* mice were also positive for YFP (Fig. 1C, D), whereas none of these cells from *ROSA-YFP* mice expressed the YFP protein. This suggests that the Nes-Cre transgene is expressed and able to activate inducible alleles not just in motor neurons but also in the majority of proprioceptive sensory neurons.

### Pan-neuronal expression of SMN arrests the loss of spinal motor neurons in a mouse model of severe SMA

**Figure 2:**
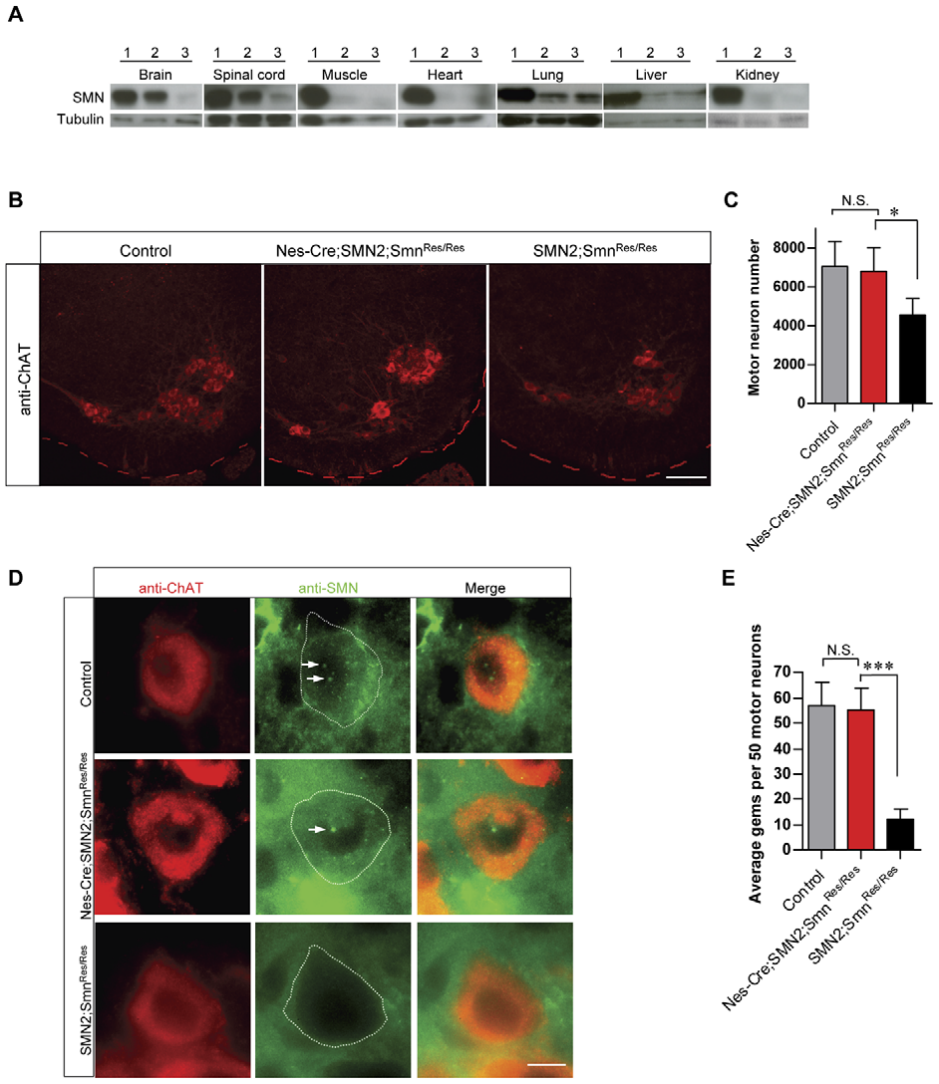
Targeted expression of SMN in neuronal tissue protects against spinal motor neuron loss in SMA mice. (**A**) Western blot analysis of tissue from PND10–12 controls (1) and SMA mice with (2) or without (3) the Nes-Cre transgene. An increase in SMN protein is observed specifically in the brains and spinal cords of SMA mice harboring the Nes-Cre transgene (lanes 2 in figure). (**B**) Immunohistochemical analysis of lumbar motor neurons of PND7 controls and SMA mice with or without the Nes-Cre transgene. Loss of motor neurons observed in the *SMN2;Smn^Res/Res^* mice is arrested in mutants expressing SMN in their neurons. (**C**) Quantification of lumbar motor neuron numbers in the above mice. *, *p*<0.05, one-way ANOVA; n≥3 mice. (**D**) Immuno-histochemical analysis of motor neurons depicting the restoration of gems (arrows) in SMA mice expressing neuronal SMN. (**E**) Quantification of gems within motor neurons of controls and mutants with or without the Nes-Cre transgene. ***, *p*<0.001, one-way ANOVA; n≥50 motor neurons. Note: Scale bars in panel (B) – 100 µm, panel (D) – 10 µm.

Having established the selective expression of the Nes-Cre transgene in nervous tissue and its relative efficiency in driving recombination in the spinal motor neurons we proceeded to use it to determine the cellular and phenotypic effects of inducing SMN protein expression from the *Smn* rescue allele in the neurons of a mouse model of severe SMA. We previously showed that the *Smn* rescue allele, prior to Cre-mediated recombination, is unable to rescue embryonic lethality of *Smn* knockout mice as it fails to produce any FL-SMN transcript in somatic tissue [28]. We also showed that 2 copies of an *SMN2* transgene are sufficient to rescue the embryonic lethality of *Smn^−/−^* knockouts and result in a mouse model of severe SMA [28], [32]. As expected, such mice *(SMN2;Smn^Res/Res^)* expressed minimal levels of the SMN protein in all tissues examined relative to controls *(Nes-Cre;SMN2;Smn^Res/+^)* carrying one copy of a wild-type murine *Smn* allele and the Nestin-Cre transgene (Fig. 2A). The heterozygotes were subsequently used as controls in all our remaining analyses and will be referred to henceforth as “controls” (Ctrls). Consistent with our RT-PCR analysis indicating selective activation of the rescue allele in nervous tissue of animals co-expressing the Nes-Cre transgene, we found that that the SMN protein was restored specifically to the brain and spinal cord tissue of *Nes-Cre;SMN2;Smn^Res/Res^* animals (Fig. 2A). In the other tissues examined, there was no difference in protein levels between the SMA mutants with or without the Nes-Cre transgene (Fig. S1).

Loss of spinal motor neurons is a hallmark of spinal muscular atrophy and has been previously observed in severely affected SMA model mice [32]. We investigated the effect of restoring SMN to the spinal neurons of SMA mice by quantifying lumbar (L1–L3) motor neurons in post-natal day (PND7) *Nes-Cre;SMN2;Smn^Res/Res^* animals and controls. Interestingly, we found that restoring SMN to the spinal neurons of SMA mice arrested the loss of large choline acetyltransferase (ChAT) positive motor neurons lying within the anterior horns of the spinal cord. Whereas *SMN2;SMN^Res/Res^* animals had ∼35% fewer motor neurons than controls *(Nes-Cre;SMN2;Smn^Res/+^)*, there was no significant difference in motor neuron numbers between the controls and *Nes-Cre;SMN2;Smn^Res/Res^* animals (Figs. 2B, C). Additionally, we observed that gems, sub-nuclear foci that contain the SMN protein and are dramatically depleted in cells from SMA patients and mouse models of the disease, were restored to the motor neurons of mutants expressing neuronal SMN (Fig. 2D, E). Together, these results suggest that restoring SMN protein within motor neurons and/or to neighboring spinal neurons is sufficient to prevent motor neuron cell bodies from degenerating as observed in SMA.

### Neuronal SMN expression mitigates central and peripheral synaptic defects of SMA model mice

**Figure 3:**
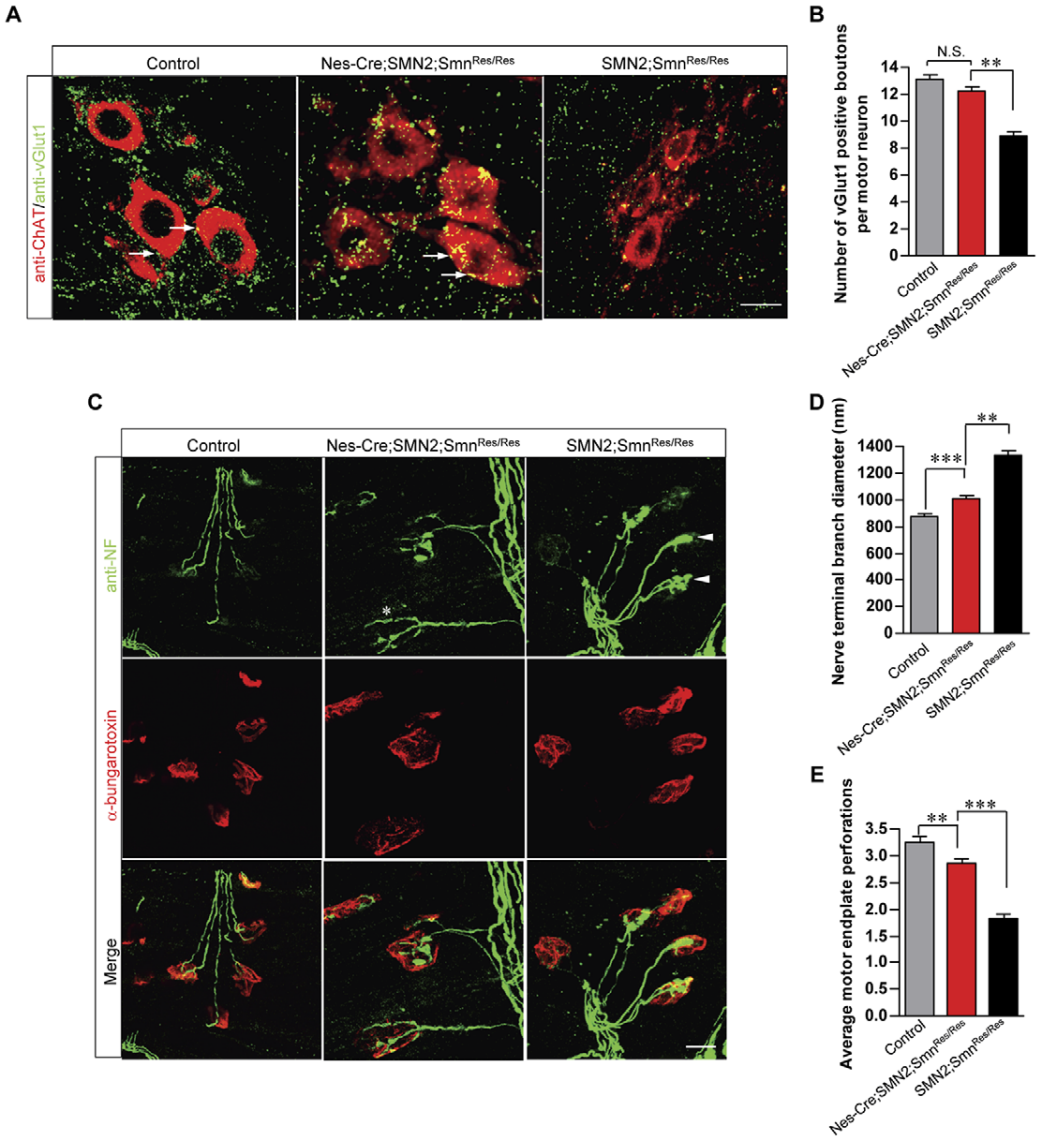
Neuronal SMN expression mitigates central and peripheral synaptic defects in severe SMA mice. (**A**) Immunostaining and (**B**) quantification of proprioceptive vGlut1 positive boutons (arrows) on the lumbar (L1–L3) spinal motor neurons of PND7 controls and SMA mice with or without high levels of SMN in their neurons. Normal numbers of Ia synapses were observed on the motor neurons of mutants expressing neuronal SMN. (**C**) Whole-mount NMJ analysis of control and SMA mice with or without normal levels of neuronal SMN. Depicted are nerve terminals (arrowheads) from *SMN2;Smn^Res/Res^* mice, swollen with neurofilaments, and one (asterix) from the *Nes-Cre;SMN2;Smn^Res/Res^* mouse that appears relatively normal. Quantification of (**D**) pre-synaptic NMJ defects as measured by the levels of neurofilament in nerve terminals which alter terminal branch diameter and, (**E**) post-synaptic NMJ defects as assessed by the complexity of motor endplates. Note: **, *p*<0.01; ***, *p*<0.001, one-way ANOVA; n>50 NMJs and n>120 motor neurons from each of 3 mice of each genotype. Scale bars in panels (A) and (C) – 20 µm.

It has become increasingly apparent that central synapses involving spinal motor neurons and proprioceptive sensory neurons, and peripheral synapses involving the motor neurons and muscle are profoundly affected in SMA models [21]–[23], [33]–[35]. Considering the protective effect on the motor neuron cell bodies of selectively expressing SMN protein in all neurons of SMA mice, we explored the possibility that central and peripheral synapses involving the spinal motor neurons of the mice were similarly protected. To examine synapses on the motor neurons, we quantified the number of proprioceptive vGlut1 positive boutons juxtaposed against the cell soma. vGlut1 positive bouton numbers were compared between *SMN2;Smn^Res/Res^* mice with or without the Nes-Cre transgene and littermate controls. As expected, significantly fewer vGlut1 positive boutons were found abutting the motor neurons of *SMN2;Smn^Res/Res^* mice relative to those on the cells of heterozygote controls. However, expressing SMN neuronally in the SMA mice restored the numbers of these proprioceptive synapses on the motor neurons to those observed in littermate controls (Figs. 3A, B), extending the protective effect of neuronally expressed SMN to sensory-motor connections. Bouton size correlates with synaptic efficacy [36]. We therefore also estimated vGlut1 bouton size on the motor neurons of the three sets of mice. vGlut1 boutons in *SMN2;Smn^Res/Res^* mice were smaller than those in controls, but increased significantly in size, though not to control levels, upon expressing SMN in the neurons of the mutants (Bouton size in µm^2^ – *SMN2;Smn^Res/Res^*: 0.50±0.01, *Nes-Cre;SMN2;Smn^Res/Res^*: 0.99±0.01, *Ctrl*: 1.13±0.02; n>600 boutons from each genotype; *p*<0.001 for each comparison, one-way ANOVA). This result suggests that expressing SMN in the neurons of SMA mice restores not just the number of vGlut1 synapses on the motor neurons but very likely their function as well.

To assess the neuromuscular synapses we examined the pre- and post-synaptic specializations in the gastrocnemious muscles of the three sets of mice at PND12–14 with an antibody against the neurofilament protein and labeled α-bungarotoxin respectively. The pre-synapses of severely affected SMA mice have previously been shown to become engorged with abnormal levels of neurofilament protein [33]–[35]. Consistent with these reports, nerve terminals of *SMN2;Smn^Res/Res^* mice appeared swollen with neurofilaments relative to those of control littermates (Fig. 3C). Accordingly, terminal branch diameter was significantly greater in the SMA mutants (Fig. 3D). Expressing SMN in the neurons of mutant mice significantly reduced neurofilament accumulation although not to levels observed in the heterozygote controls. An analysis of the more proximally located triceps muscle produced similar results (Fig. S3A). Low SMN levels have also been shown to affect the post-synapse [33]–[35] resulting, predictably, in motor endplates of reduced size and complexity in *SMN2;Smn^Res/Res^* mice (Figs. 3C, E). Expressing SMN in the neurons of the mice increased not only the size of the motor endplates but also their complexity. When the overall size, which is generally reflective of muscle fiber diameter, of the three sets of animals was taken into account, the difference in motor endplate size was no longer apparent (data not shown). However, post-synaptic complexity, a measure of NMJ maturity determined by perforations in the acetylcholine receptor clusters, unequivocally increased in the *Nes-Cre;SMN2;Smn^Res/Res^* animals in comparison to the *SMN2;Smn^Res/Res^* mutants (Fig. 3E). A complete rescue of the post-synapse was not observed as the *Nes-Cre;SMN2;Smn^Res/Res^* motor endplates remained less complex than those of the control animals. The effect of neuronal SMN on the complexity of the motor endplates was also confirmed in the triceps muscle of the three cohorts of mice (Fig. S3B). Collectively these results suggest that restoring SMN to the spinal neurons is sufficient to not only protect central synapses on the motor neurons but also those formed peripherally at the neuromuscular junctions. Interestingly, the degree of rescue (75–85%) is similar to the recombination detected at the *Smn* rescue allele locus and, perhaps reflects the number of motor neurons that were rescued following Cre expression.

### Neuronal SMN expression improves motor performance and extends survival of SMA model mice

**Figure 4:**
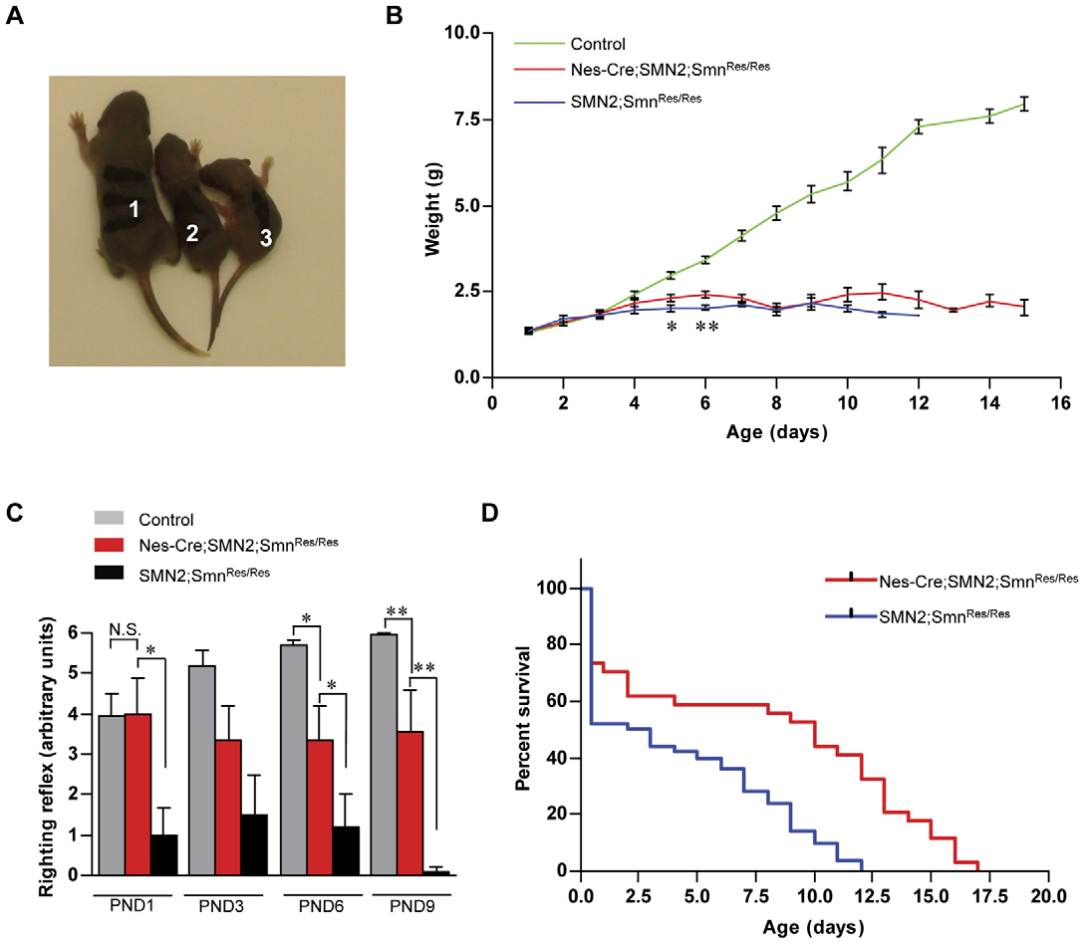
A partial phenotypic rescue of SMA mice augmented with the SMN protein in neuronal tissue. (**A**) Gross phenotypes of PND4 control (1) and SMA mice with (2) or without (3) the Nes-Cre transgene depicting a readily apparent size difference between the mutants and their normal littermate. (**B**) Weight curves of the three groups of mice highlighting the development at PND5 of a significant, albeit temporary difference between the mutants with and without an increase in the expression of SMN within the neurons. *, *p*<0.05 and **, *p*<0.01, *t* test, n>15 mice. (**C**) Expressing SMN within the neurons of SMA mice restored their ability to perform as well as controls at PND1. Additionally, motor skills were better than in littermates expressing ubiquitously low SMN at PND1, PND6 and PND9. *, *p*<0.05; **, *p*<0.01, one-way ANOVA, n≥7 mice of each genotype at all ages examined. (**D**) A comparison of lifespans, as assessed by Kaplan-Meier survival analysis, of SMA mice with or without enhanced levels of SMN protein within their neurons. Restoring SMN to the neurons of SMA mice boosted survival four-fold relative to that in littermates in which the rescue allele was not activated. *p*<0.0001, log-rank test, n≥34 mice.

Motor performance is severely impaired and lifespan greatly reduced in mouse models of SMA carrying 2 copies of the *SMN2* gene [32], [37]. Given the protective effect on the motor unit of expressing SMN in the neurons of SMA mice, we next determined if animals manipulated in this manner also exhibited a general improvement in the gross phenotype that characterizes severe mutants. *SMN2;Smn^Res/Res^* mutants with or without the Nes-Cre transgene could not be distinguished from each other or from control littermates at birth as determined by size and weight. However, by PND4, a significant difference in weight became apparent between the two mutants on the one hand and the controls on the other (Mean weight in grams – *SMN2;Smn^Res/Res^*: 1.94±0.10, *Nes-Cre;SMN2;Smn^Res/Res^*: 2.11±0.09, *Ctrl*: 2.62±0.15; n>14 for each genotype; *p*<0.05 for controls versus either mutant; *p*>0.05 when the two groups of mutants were compared, one-way ANOVA) and this persisted for the remaining duration of our analysis (also see Figs. 4A, B). Consistent with an improvement in neuromuscular pathology in the *Nes-Cre;SMN2;Smn^Res/Res^* mice, a statistically significant difference in weight between the two sets of mutants appeared at PND5. However, this disparity was temporary, eventually disappearing 48 hours later (Fig. 4B), and is indicative of only a transient effect on body weight of restoring SMN to the neurons of SMA mice.

To assess muscle strength, we subjected the mice to a well-established motor performance task – the righting reflex assay [38], [39]. Despite their equivalent sizes, *SMN2;Smn^Res/Res^* mutants performed less well at PND1 than either *Nes-Cre;SMN2;Smn^Res/Res^* pups or their control littermates (Fig. 4C). Moreover, we found that PND1 mutants expressing neuronal SMN performed as well as littermate controls. This ability eroded with time so that by PND6, *Nes-Cre;SMN2;Smn^Res/Res^* were significantly weaker than controls. Nevertheless, they performed more robustly than *SMN2;Smn^Res/Res^* mutants, a trend that continued through PND9. Results from a second test of motor performance, the hanging tube test [39] confirmed the beneficial effect of expressing SMN in the neurons of SMA mutants (Fig. S2). Together, these results suggest that expressing SMN in the neurons of severely affected SMA mice has a mitigating effect on the profound muscle weakness that characterizes mutant animals and is consistent with the reduced pathology of the motor neurons we observed in the modified mice. The results are also indicative of enhanced neuromuscular function at the NMJs of the SMA mutants expressing neuronal SMN.

Finally, to establish whether protection of the motor neurons and improved motor performance of the *Nes-Cre;SMN2;Smn^Res/Res^* mice resulted in extended lifespans of the mutant animals, we monitored their survival and compared it to that of their *SMN2;Smn^Res/Res^* littermates. Whereas the *SMN2;Smn^Res/Res^* animals displayed a median and maximum survival of 2.5 days and 12 days respectively, mutants expressing SMN in their neurons exhibited a four-fold increase in median survival to 10 days and a maximum lifespan of 17 days (Fig. 4D). Eventual death of the *Nes-Cre;SMN2;Smn^Res/Res^* mice was accompanied by a decline in movement and drop in weight. These results indicate that restoring SMN to the spinal neurons of severe SMA mice does indeed prolong survival but is insufficient to offset the effects of low SMN protein elsewhere within the animals.

## Discussion

There is an unequivocal, selective detrimental effect of ubiquitous low levels of the SMN protein on the spinal motor neurons of SMA patients and animal models. Spinal motor neurons are also predominantly affected in two other neurodegenerative diseases, amyotrophic lateral sclerosis (ALS) and spinal muscular atrophy with respiratory distress (SMARD1) [40]–[42]. However, the recognition that the pathologies of ALS and SMARD1 are the likely result of disease effects emanating from non-neuronal cells such as astrocytes [43]–[45] and myocytes respectively [46] has raised the possibility of similar parallels in spinal muscular atrophy. Accordingly, investigating the relative contributing effects of low SMN in spinal motor neurons to the SMA phenotype and the possible role of other cell types to the overall disease has gradually gained priority within the scientific and clinical communities. Considering the promise and feasibility of targeted modulation of the *SMN2* gene as a potential therapy [47], this is not surprising. For example, histone deacetylase (HDAC) inhibitors, compounds shown to promote the expression of a number of genes including *SMN2* have already been entered into clinical trials for SMA [47 and references therein]. In this report, we have built upon previous studies from our laboratory [21] and those of others [14], [19], [20] to refine the critical cellular sites of action of the SMN protein. To do so, we exploited a well-established mouse model of severe SMA and a recently constructed murine inducible *Smn* “rescue” allele to determine the consequences of selectively restoring SMN to mutant neurons, a population of cells that includes spinal motor neurons, their neighboring interneurons as well as proprioceptive sensory neurons. The last of these have emerged as possible determinants of the SMA phenotype [22], [23]. Our salient findings are as follows: First, expressing the *Smn* rescue allele following its selective, neuronal activation by the Nes-Cre transgene restores high levels of SMN to nervous tissue, including the large motor neurons of the anterior horns of the spinal cords of SMA mice. Second, restoring SMN protein to the spinal neurons of the mutant mice prevented motor neuron death and the loss of proprioceptive synapses on the motor neuron cell bodies. Third, we found that SMA mice expressing neuronal SMN exhibited significantly fewer distal defects of the neuromuscular synapses. These synapses display early and profound abnormalities of the nerve terminals as well as motor endplates in the absence of sufficient SMN. Finally, restoring SMN to SMA neurons improved motor performance and extended the lifespans of mutant mice. Nevertheless, the effects were modest relative to the level of phenotypic rescue previously achieved following ubiquitous SMN expression [48].

Notwithstanding mounting evidencing for defects of multiple organ systems in severe SMA [49]–[54], the predominantly neuromuscular phenotype arising from depletion of the ubiquitously expressed SMN protein has focused understandable attention on the disease-causing effects of low SMN in motor neurons and muscle. These effects have been investigated by both tissue-specific SMN depletion [21], [55], [56] as well as by selectively restoring the protein to SMA models [14], [19], [20], [57]–[59]. The results can be variously interpreted and depend greatly on the precise nature and reported fidelities of the experimental systems utilized. Still, based on the current literature, a general consensus emerges that implicates motor neurons as critical determinants of the SMA phenotype. Selectively depleting SMN in progenitors from which these cells arise causes a classic SMA-like phenotype, albeit one that is decidedly modest compared to the outcome of ubiquitous protein depletion [21]. Perhaps not surprisingly, restoring SMN either pan-neuronally with antisense oligonucleotides (ASOs) [57], [59] and viral vectors [58] or more specifically to motor neurons transgenically [19], [20] resulted in partial phenotypic rescue. Two studies have demonstrated more remarkable phenotypic rescue of mouse models of severe SMA [14], [60]. However, one of these studies was confounded by leaky transgene expression resulting in SMN being restored to neuronal as well as extra-neuronal tissue [14]. In the other study, an inability to sustain SMN expression in neurons of SMA mice eventually led to their premature demise limiting one's ability to draw precise conclusions of the sufficiency of selective neuronal SMN restoration to fully rescue the disease phenotype [60].

Our findings agree with the consensus [19], [20], [57]–[59] and are also consistent with a previous study in which we selectively depleted SMN in motor neurons [21]. Viewed in aggregate, these results suggest that restoring SMN to SMA motor neurons does indeed provide therapeutic benefit. However, restoring the protein to other cell types including, but perhaps not limited to the heart [52]–[54] also appears important. The modest overall rescue of the SMA mice that we observed is a likely consequence of persistent low levels of SMN outside the nervous system. A second possibility, inherent in the use of transgenic Cre drivers, is the less than perfect recombination attained in the various neurons we examined. However, this is unlikely given the inconsistency between the relatively robust expression of Cre in the neurons of SMA mutants harboring the Nes-Cre transgene and the rather muted overall therapeutic benefit observed. Other spinal neurons, and especially proprioceptive DRG neurons which either fail to form or maintain synapses on SMA spinal motor neurons could constitute yet another critical cellular site of action of the SMN protein. While determining this with certainty will require targeted depletion of the protein in sensory neurons of *SMN2* expressing models, our results and those of Gogliotti et al [19] predict that such a manipulation will likely be of little phenotypic consequence. Indeed, depleting SMN selectively in motor neuron progenitors is sufficient to precipitate motor neuron degeneration as well as a loss of vGlut1 boutons [21]. Conversely, restoring SMN selectively in motor neurons reinstates normal numbers of proprioceptive synapses on the motor neurons [19], [20]. These results suggest that the disruption of proprioceptive synapses in SMA occur cell autonomously as a consequence of reduced SMN within motor neurons. While it cannot be ruled out that reduced SMN in the proprioceptive DRG neurons exacerbates a cellular phenotype that can be driven in an entirely motor neuronal cell-autonomous manner, our current study in which we restore SMN pan-neuronally in SMA mice argues against significant therapeutic benefit following restricted SMN expression in the sensory neurons.

In conclusion, we have demonstrated that although targeted expression of SMN to nervous tissue attenuates motor neuron pathology, the mitigating effects on the overall disease are modest. Notwithstanding the caveats inherently associated with recombination efficiencies at lox P sites, our results are indicative of a critical extra-neuronal requirement for the SMN protein and argue for a systemic restoration of the protein as a means to a truly effective treatment for severe spinal muscular atrophy.

## Materials and Methods

### Mice

Nes-Cre (Jax stock # 003771) and ROSA26-flox-STOP-flox (Jax stock # 006146) mice were obtained from the Jackson Laboratory and were genotyped with primers ED-18 and ED-19 [21] and as described on the vendor's website (www.jax.org) respectively. Mice harboring the rescue (Res) allele and low copy *SMN2* transgenes respectively have been previously described [28], [32] and were interbred to generate *SMN2^+/+^;Smn^Res/+^* carriers from which mutants were derived. A forward primer HyGNI1F (5′ CCA CAT CTC CCA ACA CGA G 3′) was used with reverse primers HyGNI1RT (5′ TGC GTG TGC AAC CAG TTA AG 3′) and HyGNI1Rm ( 5′ GACAAG CTG ACA ACC ACA CG 3′) to amplify 529 bp and 489 bp fragments respectively that distinguish the hybrid rescue allele from wild-type murine *Smn*. The presence of the *SMN2* transgene was detected based on its integration site into the mouse genome as previously described [61]. The *SMN2* and Nes-Cre transgenes were maintained in the homozygous and hemizygous state respectively. Male and female mice were used for analysis and generated from carriers *(Nes-Cre;SMN2;Smn^Res/+^ x SMN2;Smn^Res/+^)* after ensuring that the Nes-Cre transgene does not effect recombination in the germline. All animal procedures were performed according to institutional guidelines.

### RT-PCR and western blot analysis

To test the tissue specificity with which the Nes-Cre transgene effected recombination of the rescue allele, tissue from PND7 *Nes-Cre;Smn^Res/+^* mice was harvested, RNA extracted with Trizol (Invitrogen) and cDNA prepared according to established protocols. Primers mSMN exon6 For (5′ GTC TGG ATG ACA CTG ATG CCC 3′) and Exon8ISH Rev (5′ CAA TGA ACA GCCATG TCC AC 3′) were used to amplify a 681 bp fragment indicating the presence of FL-SMN, or a 627 bp fragment indicating the presence of the SMNΔ7 transcript. SMN protein levels in PND10–12 end-stage (defined as 2 consecutive days of weight loss, or a ≥15% loss in bodyweight, whichever came first) SMA mutants and controls were determined by western blot analysis as previously described [32]. We used the following antibodies for our analysis: anti-SMN (1∶10,000, BD Biosciences), anti-α-tubulin (1∶5000, Sigma Aldrich) and an HRP-linked IgG (1∶10,000, Jackson Immunoresearch) secondary antibody which was visualized using the ECL Plus western blotting kit (GE Healthcare).

### Immunohistochemistry

NMJs were analyzed in the gastrocnemius and triceps muscles. Prior to tissue extraction, mice were perfused with 1× PBS and muscles stored at −80°C until they were analyzed. For analysis, the muscle was incubated in ice-cold methanol (3 mins), and then teased to remove the fascia before being rinsed in ice-cold 1× PBS (10 mins). Samples were then incubated in blocking solution [3% bovine serum albumin (FBS), 1% Triton X-100 in PBS] overnight at 4°C to reduce non-specific binding of antibodies and treated with an anti-neurofilament (NF) antibody (1∶1000, Millipore) for 2 days (4°C). After washing, the samples were incubated with Alexa 488-conjugated secondary antibody (1∶500, Invitrogen) and Alexa-594 conjugated α-bungarotoxin (1∶1000, Invitrogen). Excess antibody was washed off with PBS-T, samples mounted in Vectashield (Vector Labs) and NMJs imaged on a Leica TCS SP5 II confocal microscope (Leica Microsystems). Pre-synaptic defects were quantified by determining the nerve terminal branch diameter. Specifically, the axon terminal was followed until it branched in the NMJ for the first time, and the widest portion of the branch in the distal half measured to determine levels of NF accumulation. Post-synaptic complexity was determined by the number of perforations per motor end-plate. Only *en face* endplates were included in the quantification. Perforations were defined as “holes”, areas devoid of staining circumscribed by regions that either stained intensely or diffusely with labeled α-bungarotoxin.

To quantify motor neurons and examine Ia synapses on them, mice were perfused with 1× PBS and 4% paraformaldehyde (PFA), spinal cords dissected, post-fixed in 4% PFA (2 hrs) and cryoprotected in sucrose for 2 days (4°C). Each cord was then separated into lumbar, thoracic and cervical segments, embedded in OTC medium (VWR) and 20 µm thick transverse sections cut on a Leica 3050S cryostat (Leica Microsystems). The sections were spaced 200 µm apart and spanned a 3 mm region of cord from L1–L3, C6–C8 or T10–T12. For motor neuron counts the sections were stained with an anti-ChAT (1∶1000, Millipore) antibody. To quantify vGlut1 boutons on the motor neurons, sections were dual labeled with the ChAT antibody and one that recognizes vGlut1 (Chemicon, 1∶500). Secondary antibodies used were: FITC-conjugated anti-guinea pig (1∶500, Jackson Immunoresearch), Alexa-488 conjugated donkey anti-goat (1∶500, Invitrogen). Sections were visualized and imaged on either a conventional fluorescent (Nikon E80i) or confocal microscope as described for the NMJs. To ensure consistency between samples, vGlut1 boutons were counted only in sections where the motor neuron nucleus was clearly visible. Furthermore, only puncta abutting the periphery of the motor neuron cell bodies were counted.

The specificity and efficiency of Nes-Cre-mediated recombination was tested in PND14 *Nes-Cre;ROSA26* and PND7 *Nes-Cre;SMN2;Smn^Res/Res^* mice and relevant controls as described in the results section. Mice were perfused as described above. Spinal cords with DRGs intact were dissected and 20 µm sections from lumbar, thoracic and cervical regions prepared. Prior to treatment with antibodies, sections were post-fixed in 4% PFA (10 mins), washed in 1× PBS, permeabilized (0.5% Triton X-100 in PBS) and finally incubated in blocking medium (10% FBS, 0.2% Triton X-100 in PBS). To detect YFP expressing cells and motor neurons, cryosections were stained with primary antibodies specific for green fluorescent protein (GFP) (1∶1000, Invitrogen) and ChAT (1∶200, Millipore) followed by corresponding secondary antibodies: donkey anti-rabbit Alexa-488 (1∶2,000, Invitrogen) and donkey anti- goat Alexa-594 (1∶2000, Invitrogen). DRG neurons were stained with an antibody against GFP as described above and an antibody against parvalbumin (1∶2000, gift from the Jessell lab). SMN-containing gems were detected with a 1∶500 dilution of the antibody used for the western blots above and a corresponding secondary (donkey anti-mouse Alexa-488; 1∶1000, Invitrogen). All primary antibody incubations were carried out overnight at 4°C and secondary antibody incubations for 2 hours at room temperature. Antibody solutions contained 2.5% FBS, 0.2%Triton X-100 in PBS and all washes were done using 0.1%Triton X-100 in PBS. Developed slides were overlaid with liquid mounting media Vectashield (Vector Labolatories). Fluorescent images were acquired using either an E80i Nikon microscope equipped with a 20× objective and Spot Flex digital camera (Diagnostic Instruments) or a Leica TCS SP5 II confocal microscope equipped with a 63× objective.

### Gross phenotypic assays

Righting reflex was used to estimate muscle strength in mutants and control littermates as described previously [38]. Mice were placed on their backs, and latency to turn over and place all four paws on the bench top was recorded. The following arbitrary scores were used to quantify impaired righting ability: 6 for 0–5 s, 5 for 5–9 s, 4 for 10–14 s, 3 for 15–19 s, 2 for 20–24 s, 1 for 25–30 s, and 0 if >30 s. The procedure was repeated thrice for each subject, and the best performance recorded as the righting ability score. The hanging tube test was performed as previously described [39]. The length of time the pups were able to remain suspended from the rim of the 50 ml tube was recorded. Body weights were recorded from PND1 onwards. For survival curve analysis, all animals found dead on the day of birth were assumed to have been born alive and succumbed to disease on PND0.

### Statistics

All statistical analysis was performed using GraphPad Prism version 4.0 (GraphPad Software). Kaplan-Meier survival curves were compared using the log-rank test equivalent to the Mantel-Haenszel test. To compare means for statistical difference, either the Student's *t* test or one-way ANOVA followed by Tukey's multiple comparison test was performed. Data in the manuscript are presented as mean ± SEM unless otherwise indicated. Values of *p*<0.05 were considered significant.

## Supporting Information

Figure S1 **Quantification of protein levels in controls and SMA mutants with or without the Nes-Cre transgene.** Bands from corresponding tissues of the various cohorts were averaged and compared with their respective loading controls. The normalized averages were compared to their respective controls and plotted graphically. Note: n≥3 independent experiments. **, *p*<0.01; *** *p*<0.001, one-way ANOVA.
(TIF)

Figure S2 **SMA mice expressing neuronal SMN exhibit improved motor performance in the hanging tube test.** Note: n≥10 for each cohort of mice. *, *p*<0.05; ** *p*<0.01, one-way ANOVA.
(TIF)

Figure S3 **Neuronal SMN attenuates NMJ pathology at the pre- as well as post-synapses of the triceps muscle.** (**A**) Quantification of pre-synaptic NMJ pathology as assessed by neurofilament accumulation in the nerve terminals. (**B**) Quantification of post-synaptic NMJ pathology as assessed by the complexity of the motor endplates. Note: n≥100 NMJs for each genotype. *, *p*<0.05, one-way ANOVA.
(TIF)
