# Supplementary figures and images for: Limited Phenotypic Effects of Selectively Augmenting the SMN Protein in the Neurons of a Mouse Model of Severe Spinal Muscular Atrophy
Source: PLoS One. 2012 Sep 27;7(9):e46353. doi: 10.1371/journal.pone.0046353 (PMC3459898; doi:10.1371/journal.pone.0046353)

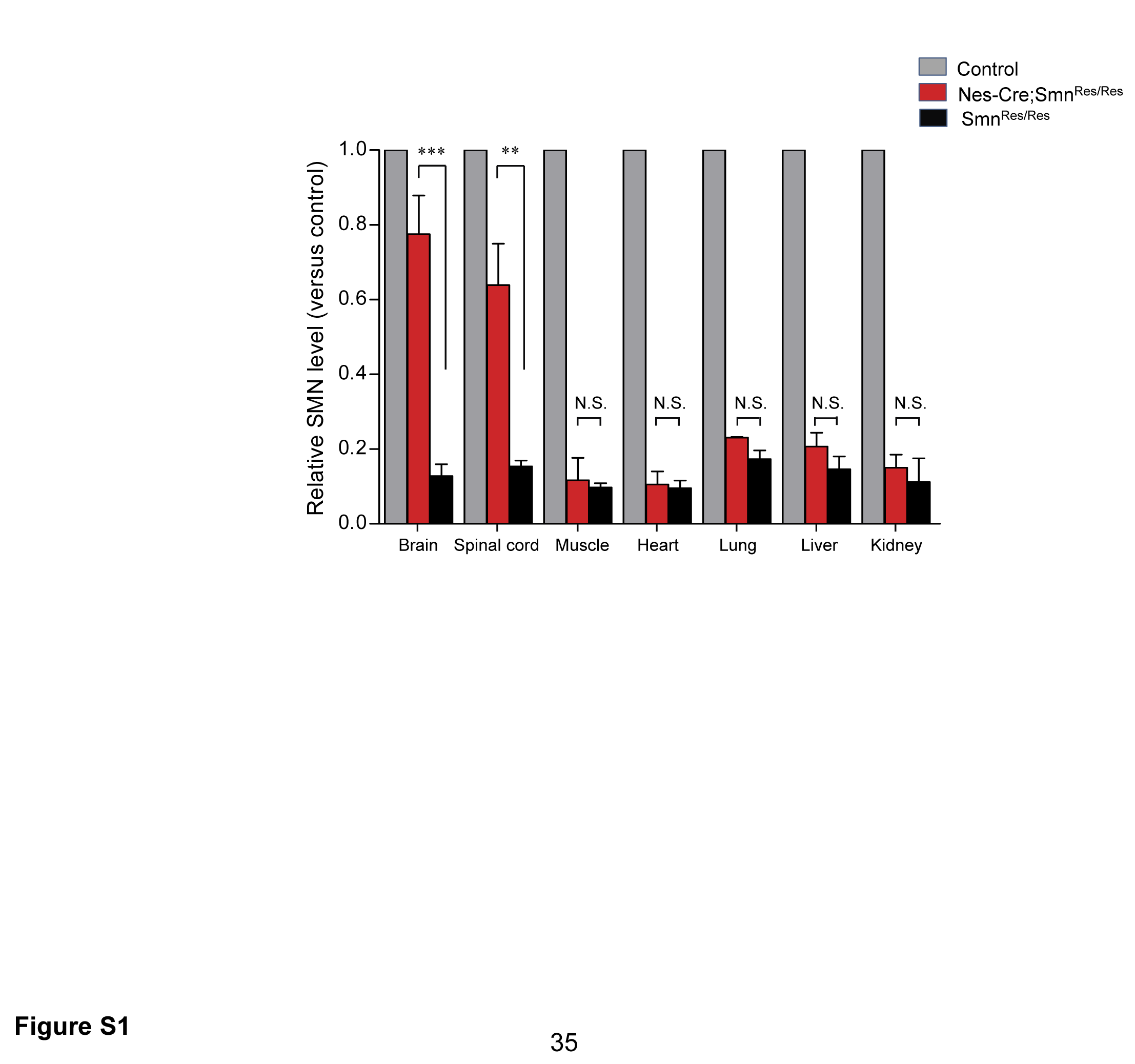

Supplement: Figure S1 — Quantification of protein levels in controls and SMA mutants with or without the Nes-Cre transgene. Bands from corresponding tissues of the various cohorts were averaged and compared with their respective loading controls. The normalized averages were compared to their respective controls and plotted graphically. Note: n≥3 independent experiments. **, p<0.01; *** p<0.001, one-way ANOVA. (TIF) [file pone.0046353.s001.tif]

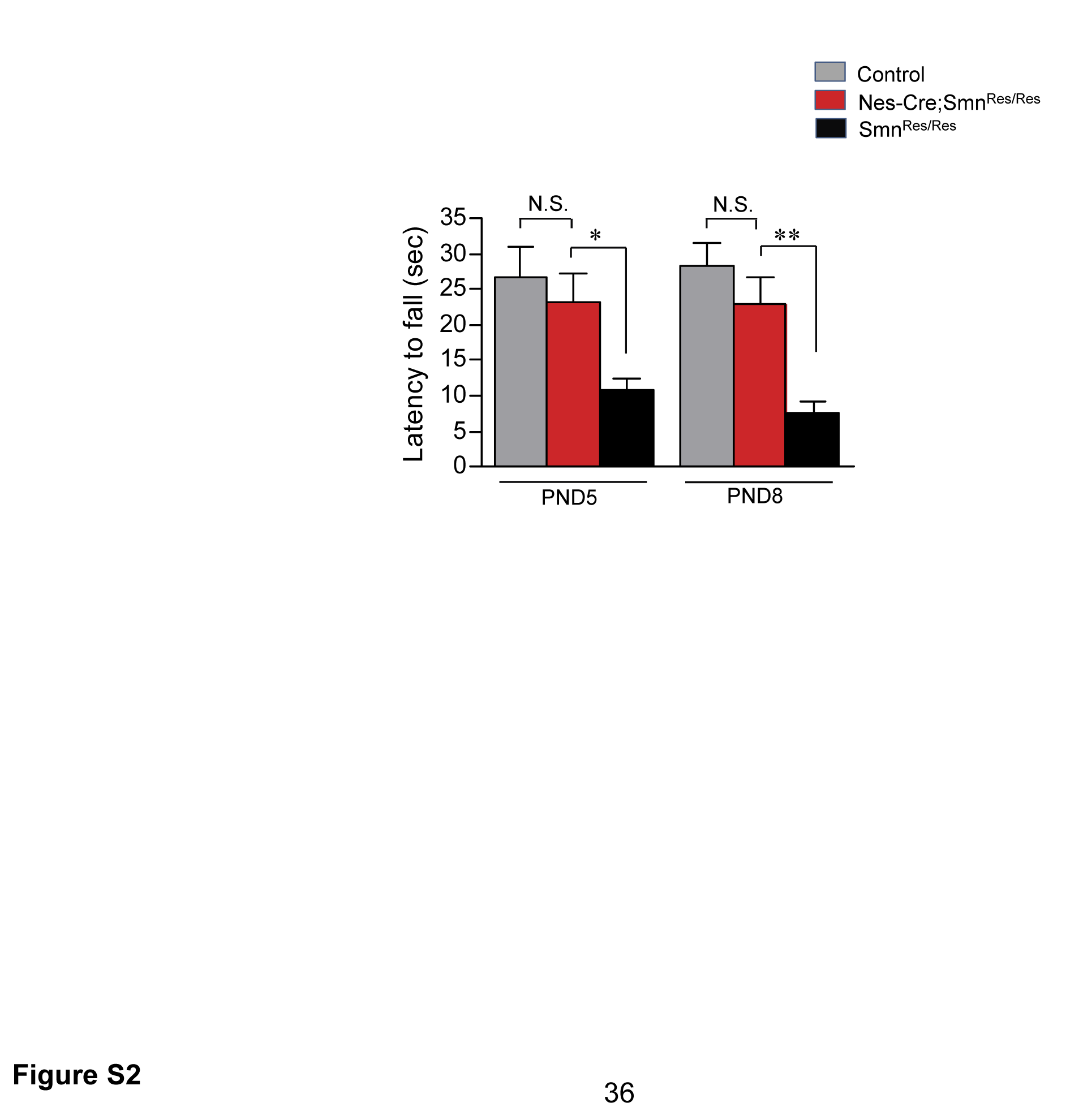

Supplement: Figure S2 — SMA mice expressing neuronal SMN exhibit improved motor performance in the hanging tube test. Note: n≥10 for each cohort of mice. *, p<0.05; ** p<0.01, one-way ANOVA. (TIF) [file pone.0046353.s002.tif]

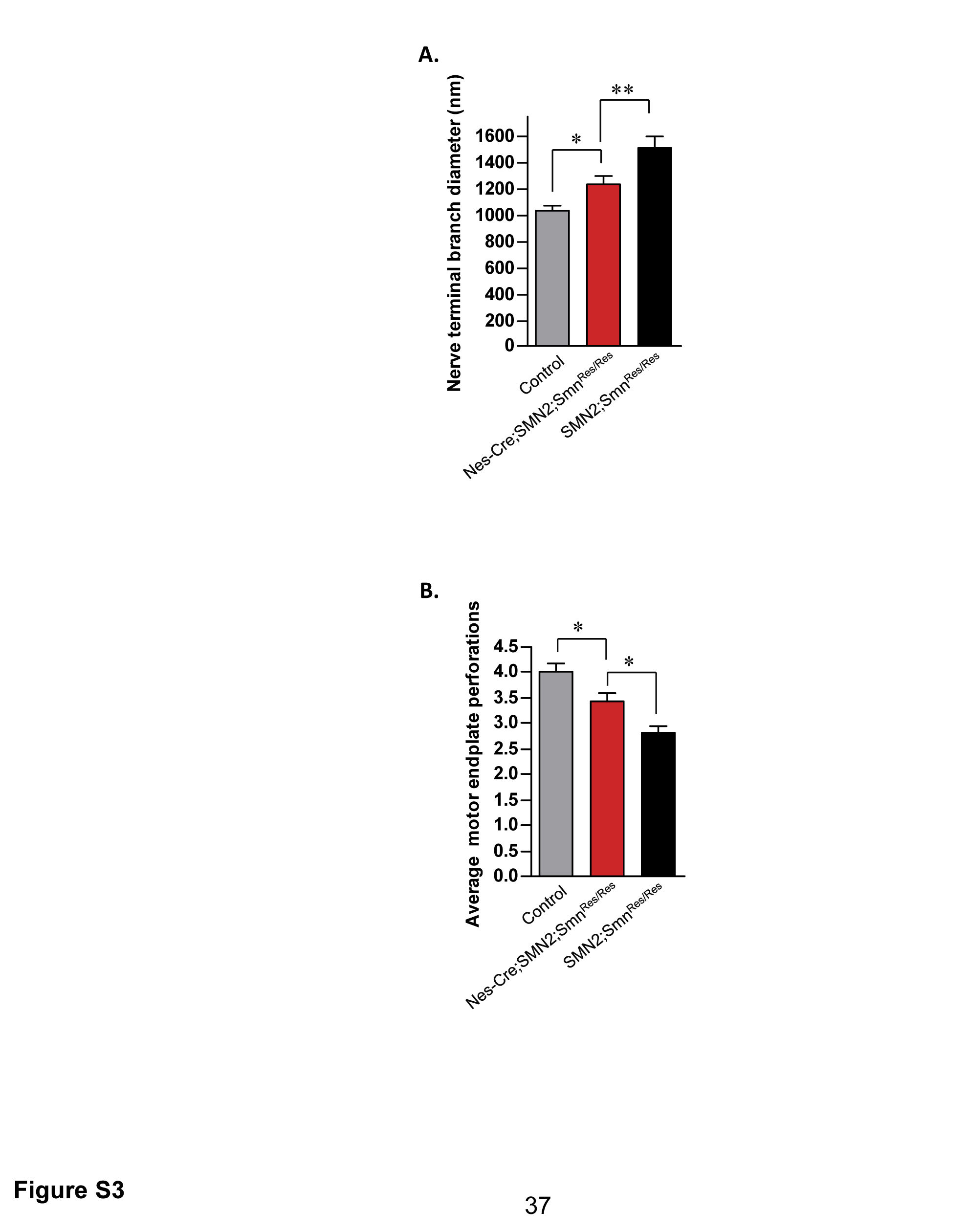

Supplement: Figure S3 — Neuronal SMN attenuates NMJ pathology at the pre- as well as post-synapses of the triceps muscle. (A) Quantification of pre-synaptic NMJ pathology as assessed by neurofilament accumulation in the nerve terminals. (B) Quantification of post-synaptic NMJ pathology as assessed by the complexity of the motor endplates. Note: n≥100 NMJs for each genotype. *, p<0.05, one-way ANOVA. (TIF) [file pone.0046353.s003.tif]
